# Reply to Ferrés-Padró et al. Comment on “Lionte et al. Association of Multiple Glycemic Parameters at Hospital Admission with Mortality and Short-Term Outcomes in Acutely Poisoned Patients. *Diagnostics* 2021, *11*, 361”

**DOI:** 10.3390/diagnostics11061032

**Published:** 2021-06-04

**Authors:** Victorita Sorodoc, Catalina Lionte, Cristina Bologa, Ovidiu Rusalim Petris, Laurentiu Sorodoc

**Affiliations:** 1 Internal Medicine and Clinical Toxicology Department, Faculty of Medicine, “Grigore T. Popa” University of Medicine and Pharmacy, 700115 Iasi, Romania; vivisorodoc@yahoo.com (V.S.); crisbologa@yahoo.com (C.B.); laurentiu.sorodoc@gmail.com (L.S.); 2 2nd Internal Medicine Clinic, “Sf. Spiridon” Emergency Clinic County Hospital, 700111 Iasi, Romania; ovidiupetris@yahoo.com; 3 Internal Medicine and Nursing Department, Faculty of Medicine, “Grigore T. Popa” University of Medicine and Pharmacy, 700115 Iasi, Romania

Thank you for the opportunity to respond to the issues raised by Ferrés-Padró et al. in their recent letter to the editor. All authors would also like to thank Dr. Ferrés-Padró and his colleagues for their recognition of our work [1]. 

First, we noticed the differences in the prevalence of different poisons in the data reported by Dr. Ferrés-Padró. A higher proportion of pesticides, toxic alcohols, and chemicals, and caustics was detected in our patients at 26.6% vs. 6.4% in their analysis. Pesticides and methanol had been associated before our study with changes in blood glucose level and mortality [2,3]. The incidence of intoxication with a combination of poisons was higher, while illicit drug poisoning was lower as compared with the data reported by Dr. Ferrés-Padró. This might explain why the differences were noticed in their analysis. 

It should also be noted that we did not analyze a poisoned pediatric population in addition to the poisoned adults we discussed in our paper. This might explain the poor results of non-invasive determination of blood glucose of adults and children poisoned or exposed to xenobiotics that they were mentioned. 

Our first retrospective study on 15,497 patients with acute poisoning showed that in non-diabetic subjects with acute poisoning, the prevalence of toxic-induced hypoglycemia depends on the poison itself, the mechanism of poisoning, and the association between toxics and the severity of toxic-induced liver disease. Furthermore, factors predicting a negative outcome in toxic-induced hypoglycemia were the association of toxins, cardiac and hepatic complications, and age (>65 years) [4]. 

We noticed a high prevalence of ethanol poisoning in the data reported by Dr. Ferrés-Padró. Our experience showed that hypoglycemia was recorded in more than two-thirds of patients acutely poisoned with ethanol, when alcohol level was 0.5–1.5 g/L, while impaired glucose tolerance was recorded in half of poisoned patients with blood ethanol levels > 2.5 g/L [5].

Large observational studies have reported increasing trends of severe hypoglycemia, resulting in emergency visits or hospitalization, in the USA, Canada, England, and Japan, possibly related to the rising prevalence of diabetes in older people [6]. We showed that the admission blood glucose is important in predicting the outcomes of the hospitalized patients acutely poisoned with xenobiotics, regardless of diabetes status [1]. 

We share the opinion of Ferrés-Padró et al., who have recently proposed the routine determination of glycemia in poisoned patients and its inclusion in the panel of healthcare quality indicators of these patients [7]. 

Emergency medical services activations for hypoglycemia are sizeable, and 41% of Emergency Department (ED) admissions for hypoglycemia arrive by ambulance [8]. However, a considerable proportion of patients are discharged from the ED, and increased odds of overnight admission include infection and acute renal failure [8].

Blood glucose disturbance is a common phenomenon during critical illness, can be quickly detected in the prehospital setting, and recently was proved to improve the accuracy of the National Early Warning Score (NEWS) to enhance identification of patients at risk of death [9].

Although the use of NEWS is not generalized in all European prehospital settings, together with Ferrés-Padró et al., we hope that future prospective studies in different geographical areas and by different prehospital emergency medical services and emergency departments will provide the external validation of the utility of all bedside parameters for prehospital adverse events used in the poisoned patients. 
